# Generation of Inducible Gene-Switched GAL4 Expressed in the *Drosophila* Female Germline Stem Cell Niche

**DOI:** 10.1534/g3.119.400246

**Published:** 2019-04-24

**Authors:** Yi-Teng Ke, Hwei-Jan Hsu

**Affiliations:** Institute of Cellular and Organismic Biology, Academia Sinica, Taipei 11529, Taiwan

**Keywords:** GSC, inducible *GAL4*, GS GAL4, insulin, Dilp, aging, P{Switch}

## Abstract

The stem cell niche, a regulatory microenvironment, houses and regulates stem cells for maintenance of tissues throughout an organism’s lifespan. While it is known that stem cell function declines with age, the role of niche cells in this decline is not completely understood. *Drosophila* exhibits a short lifespan with well-characterized ovarian germline stem cells (GSCs) and niche compartments, providing a good model with which to study stem cell biology. However, no inducible tools for temporal and spatial control of gene expression in the GSC-niche unit have been previously developed for aging studies. The current UAS-GAL4 systems are not ideal for aging studies because fly physiological aging may be affected by the temperature shifts used to manipulate GAL4 activity. Additionally, the actual needs of the aged niche may be masked by continuously driven gene expression. Since GeneSwitch GAL4 is conveniently activated by the steroid RU486 (mifepristone), we conducted an enhancer-trap screen to isolate GeneSwitch GAL4 lines with expression in the GSC-niche unit. We identified six lines with expression in germarial somatic cells, and two lines (#2305 and #2261) with expression in niche cap cells, the major constituent of the GSC niche. The use of lines #2305 or #2261 to overexpress *Drosophila* insulin-like peptide 2, which maintains GSC lifespan, in aged niche cap cells significantly delayed age-dependent GSC loss. These results support the notion that insulin signaling is beneficial for maintaining aged stem cells and also validate the utility of our GeneSwitch GAL4 lines for studying stem cell aging.

Adult stem cells have the capacity to undergo asymmetrical division, thereby renewing themselves and also generating differentiated daughter cells to replenish lost cells for tissue homeostasis. Stem cells reside in a microenvironment, called the niche, which provides both stemness factors and physical contact to regulate stem cell identity (Moore and Lemischka 2006). During aging, stem cell function and/or number are known to decline (Brack and Rando 2007). For example, aging decreases the muscle stem cell population by promoting differentiation and impairing regeneration efficiency (Chakkalakal *et al.*, 2012; Gopinath and Rando 2008). Similarly, aging-associated decreases in hematopoietic stem cells, which produce the lymphoid and erythroid lineages, have been shown to impair the immune system (Beerman *et al.*, 2010; Geiger *et al.*, 2013). Despite these reports of functional impairment with age, the mechanisms governing stem cell aging-related decline remain unclear.

The *Drosophila* ovary is an excellent model with which to study stem cell biology because it contains well-characterized germline stem cells (GSCs) and niche cells (Figure 1A-C) (Kirilly and Xie 2007). Each ovary is composed of 15-20 ovarioles, the functional units that produce eggs (Spradling 1993). The anterior-most structure of the ovariole is the germarium, which houses two or three GSCs in a niche that includes the terminal filament (TF), cap cells, and anterior escort cells at the anterior tip of the germarium (Kirilly and Xie 2007). GSCs form direct contacts with cap cells, the major components of the niche (Song and Xie 2002), and contain a specialized membrane-rich organelle called the fusome, which is adjacent to the GSC-cap cell interface (de Cuevas and Spradling 1998). Each GSC division gives rise to a cystoblast that undergoes four rounds incomplete division to become a 16-germ cell cyst interconnected by a branched fusome. After the 16-cell cyst is encased by a layer of follicle cells, it buds off from the germarium and further develops into a mature egg. In addition to the well-characterized nature of the *Drosophila* ovary, the relatively short lifespan and amenability to genetic approaches also make *Drosophila* an ideal organism model to study stem cell aging (Helfand and Rogina 2003). However, in order to better utilize this model, genetic tools to manipulate gene expression in the aged GSC-niche must be developed.

**Figure 1:**
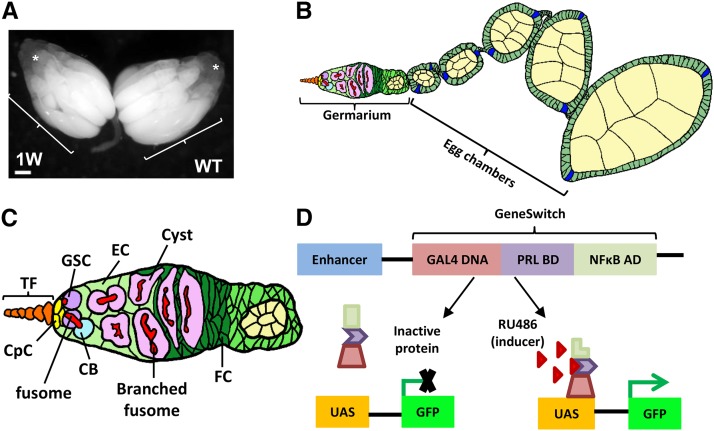
The *Drosophila* ovary and GeneSwitch GAL4. (A) A pair of one-week (W)-old ovaries connected by the oviduct; each ovary contains ∼20 ovarioles. Asterisk shows anterior pre-vitellogenic egg chambers, and arrow points to vitellogenic egg chambers. Scale bar is 50 µm. (B) The arrangement of an ovariole. The ovariole is composed of a germarium and developing egg chambers in a linear array of progressive developmental stages; young egg chambers are located closer to the germarium, while older egg chambers are positioned closer to the oviduct. (C) The scheme of the germarium. Terminal filament (TF) cells, cap cells (CpCs) and anterior escort cells (ECs) form a GSC niche to house GSCs, which possess unique organelles called fusomes. GSC immediate daughter cells, cytoblasts (CBs), undergo incomplete divisions to become germ cell cysts, which are interconnected by a branched fusome. The germ cell cyst is then surrounded by a layer of follicle cells (FCs), and buds off from the germarium to become a newly formed egg chamber. (D) The GeneSwitch GAL4 system. The chimeric gene (Gene-Switch) is under the control of a tissue specific enhancer and encodes the GAL4 DNA-binding domain, the human progesterone receptor-ligand-binding domain (PRL BD), and the activation domain (AD) of human NF-κB. Without ligand (RU486), the chimeric GAL4 is inactive. In the presence of RU486, the chimeric GAL4 is activated and binds to *UAS* to drive transgene (X) transcription.

Ovarian GSC maintenance is reduced with age (Kao *et al.*, 2015), but *UAS-GAL4*-mediated overexpression of *Drosophila* insulin-like peptide 2 (Dilp2) in the GSC niche after eclosion can delay age-dependent GSC loss (Hsu and Drummond-Barbosa 2009). Notably, continuous overexpression of Dilp2 may exceed the physiological requirement for the protein, masking the actual needs of the aged niche. To address this issue, we generated inducible GeneSwitch GAL4 lines that in the presence of mifepristone (RU486), drive *UAS* transgenes in specific temporal and spatial patterns within the GSC niche. Of the 2385 GeneSwitch GAL4 lines generated, 79 lines showed expression in the ovary. Among these 79 lines, only five expressed the reporter in germarial somatic cells, including lines #1774 (cap cells and follicle cells), #2112 (cap cells and TFs), #2305 and #2261 (cap cells, TFs and follicle cells), and #2126 and #2312 (escort cells). Line #2112 did not drive expression in the aged niche, and line #2126 had leaky expression in the absence of RU486. Importantly, overexpressing Dilp2 in the aged GSC niche with lines #2261 and #2305 delayed age-dependent GSC loss, validating the utility of these tools for studying GSC aging. Furthermore, we identified *vrille*, *Mob2*, *Uncoordinated 115a*, *Ecdysone Receptor*, *Glutathione S transferase 1* and *failed exon connections* as targeted genes in lines #1774, #2112, #2261, #2305, #2126 and #2312, respectively. Our results not only introduce inducible genetic tools for manipulating gene expression in the GSC niche, but they also identify genes that might be expressed in the GSC niche.

## Materials and Methods

### Fly strains and husbandry

Fly stocks were maintained at 22-25° on standard medium, unless otherwise indicated. *yw* was used as a wild-type control. *P{switch2}_19-2_* (B# 6849) has been previously described (Roman *et al.*, 2001) and was obtained from the Bloomington Drosophila Stock Center. *UAS-dilp2* has been previously described (Hsu and Drummond-Barbosa 2009; Pan *et al.*, 2007). Other genetic tools are described in flybase (http://flybase.org).

### RU486 administration

A 10 mg/ml stock solution of RU486 (mifepristone; Sigma) was made in pure ethanol (Sigma). Two methods were used to prepare RU486-containing food. For standard medium containing RU484, 0.5 ml of RU486 stock solution was diluted into 499.5 ml of standard medium to yield a final concentration of RU486 is 10 µg/ml. For wet yeast containing RU486, an appropriate concentration of RU486 solution was made in ddH_2_O and then added to dry yeast (55% (w/v), RED STAR) and mixed well. For the aging experiments, standard medium was substituted with molasses-agar medium to ensure RU486 uptake was only from wet yeast. Control flies were only fed with wet yeast that did not contain RU486 plus molasses-agar medium. Molasses-agar medium was prepared with 500 ml ddH_2_O, 45 ml molasses (Groeb farms), and 11 g agar (GeneTeks). The components were mixed well and microwaved until boiling, after which the mixture was cooled and 9.25 ml of 20% Tegosept (Sigma, an antifungal agent) in methanol was added.

### Egg laying assay

For egg count assay, five *yw* females (one or two-day-old) and five *yw* males were grown in a plastic bottle containing a molasses plate (50% Molasses 65.52 ml, H_2_O 176.734 ml and 8 g of agar) with a layer of wet yeast containing designed RU486, in triplicate at 29°. The molasses plate was changed daily to provide moisture, sugar and food; the replaced plate was collected for 5 days for counting egg number.

### P-element mobilization

**Figure 2:**
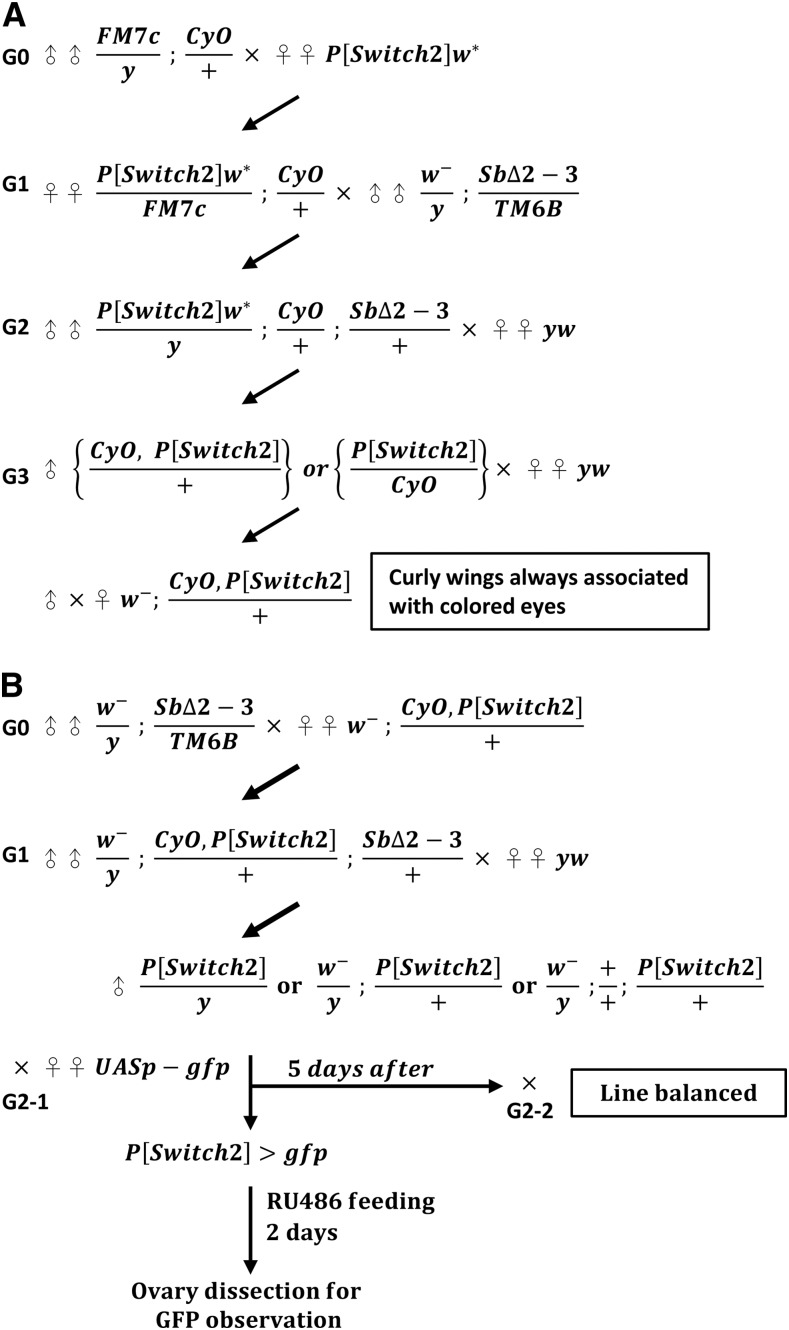
The crossing scheme used to generate flies for screening *GeneSwitch GAL4* lines with GSC-niche unit expression. (A) Generation of the new *[GeneSwitch]* on the *CyO* balancer chromosome for the large scale screen. The *p{Switch2}* line from the Bloomington stock center is located on the X chromosome, which carries a *white* (w*) gene that causes flies to have red eyes. To mobilize *p{Switch2}*, female virgins (♀) of p{Switch2}w* were crossed with male flies (♂) with the genotype *FM7c/Y*; *CyO/+* (Generation (G) 0). The progeny with the genotype of p{Switch2} w*/*FM7c* were crossed with males carrying the transposase (*w^-^/Y*; *SbΔ2-3/TM6B*) (G1). The male progeny of this cross with the genotype of p{Switch2}w*/+; *CyO/+*; *SbΔ2-3/+* (*p{Switch2}* is mobilized in the genome) were further crossed with *yw* virgins (exhibit white eyes due to mutated *w* gene) to get rid of transposase and w* (G2). The male progeny of G2 carrying *yw/Y*; *p{Switch2}*/CyO or *yw/Y*; *CyO*. *p{Switch2}* /+ were then individually crossed with *yw* virgins (G3) to distinguish their genotype. If the G3 progeny exhibited curly wings and always colored eyes, then the parental genotype must be *yw/Y*; *CyO*. *p{Switch2}* /+; *p{Switch2}* includes a mini *white* gene. The fly line with the genotype *CyO*. *p{Switch2}* /+ was used to generate *GeneSwitch GAL4* lines for screening. (B) Scheme for screening *GeneSwitch GAL4* lines expressed in the GSC-niche unit. *p{Switch2}* was mobilized in flies of the genotype *CyO*. *p{Switch2}* /+ ; *SbΔ2-3/+*, which were obtained by crossing males (*w^-^/Y*; *SbΔ2-3/TM6B*) with female virgins (*CyO*. *p{Switch2}* /+) (G0). Flies of the genotype *CyO*. *p{Switch2}* /+ ; *SbΔ2-3/+* were further crossed with *yw* female virgins (G1) to remove the transposase allele from their progeny. Individual males (*p{Switch2}*/Y, +/Y; *p{Switch2}*/+, *+/Y*; *CyO p{Switch2}* /+ or +/Y; +/+; *p{Switch2}*/+) from the G1 progeny were crossed with female virgins carrying *UASp-gfp* (G2-1). After mating, the same males were used for balancing (G2-2) by flies carrying balancers. Eclosed female progeny from the G2-1 cross (*p{Switch2}>UASp-GFP)* were fed with RU486 (10 µg/mL) for two days and their ovaries were dissected for GFP observation.

Mobilizations of the X-linked *P{Switch2}_19-2_* element were performed using the *SbΔ2-3* as a transposase source (crossing scheme is shown in Figure 2), as previously described with minor modifications (Nicholson *et al.*, 2008). New insertions of *P{Switch}_19-2_* on the *CyO* balancer chromosome were selected in the large scale screen. For inducible GAL4 line generation, males with a genotype of *w*^-^; *P{switch2}CyO/+*; *TMS SbΔ2-3/+* were crossed with *yw* female virgins. Non-*CyO* male progeny from this cross carrying *P{switch2}* were first selected for the screen. Because a low number of non-*CyO* males carrying *P{switch2}* were found, we also selected *CyO* males with eye color that had changed from red to orange or yellow, an indicator for *P{switch2}* jumping. These new *P{Switch2}* insertions were used for expression screening in the adult ovary.

### Immunohistochemistry and fluorescence microscopy

For immunostaining, ovaries were dissected, fixed and immunostained at designated ages as described previously (Tseng *et al.*, 2014). In brief, ovaries were dissected in Grace’s insect medium (Lonza) and fixed with 5.3% paraformaldehyde/Grace’s insect medium for 13 min with gentle agitation at room temperature (RT). Ovaries were washed in PBST (0.1% Triton X-100 in PBS) for 30 min three times, and teased apart in PBST, after which the tissue was incubated with blocking solution (GOAL Bio) for 3 h at RT or 4° overnight. Ovaries were incubated with primary antibodies (diluted in blocking solution) for 3 h at RT or 4° overnight, followed by three PBST washes of 30 min each. Next, ovaries were incubated with secondary antibodies (diluted in blocking solution) for 3 h at RT or 4° overnight, followed by PBST washing. The primary antibodies were as follows: mouse anti-Hts (1B1) (Drosophila Studies Hybridoma bank, DSHB, 7H9, 1:25), mouse anti-Lamin (Lam) C (DSHB LC28.26, 1:25 or 1:12.5), rabbit anti-Vasa (Santa Cruz Sc-30210, 1:250), rabbit anti-GFP (Torry Pines GTX113617, 1:1500). Samples were stained with 0.5 μg/ml DAPI (Sigma, diluted in PBST, 1:1000), mounted in 80% glycerol containing 20.0 µg/mL N-propyl gallate (Sigma), and analyzed with a Zeiss LSM 700 confocal microscope.

GSCs were identified as those cells with a fusome labeled by 1B1 antibody that were adjacent to cap cells, which were labeled by LamC (Tseng *et al.*, 2014).

### RNA extraction and quantitative real-time PCR

Total RNA was extracted from the anterior part of 20 pairs of ovaries. Twenty microliters of Trizol (Life technologies) was added to the sample before homogenization with a tissue grinder and storage at -80°. BCP (Sigma) and isopropanol (MERCK) were used for extraction. Seventy-five percent ethanol was made using DEPC (Sigma) water, and 30 µl DEPC water was used to resuspend the RNA pellet. Total RNA (1 µg) was reverse transcribed with the Transcriptor First Strand cDNA Synthesis kit (Roche). Steady-state mRNA levels were determined using the LightCycler 480 Probes Master combined with a Universal ProbeLibrary (Roche). The probes and primer sets for each gene were designed from the Roche Universal ProbeLibrary assay design center (https://lifescience.roche.com/global_en.message.html#reference-gene-assays) and are listed below.

*dilp2*: probe #63, 5′-CTCAATCCCCTGCAGTTTGT-3′ and 5′-GCGGTTCCGATATCGAGTT-3′
*RpL19*: probe #128, 5′-GAGCGTATTGCCACCAGGA-3′ and 5′- CGATCTCGTCCTCCTTAGCA-3′;

### Splinkerette PCR

Splinkerette PCR was performed as previously described (Potter and Luo 2010). Total genomic DNA was extracted from 10 flies using the DNeasy Blood & Tissue kit (QIAGEN). One microgram of genomic DNA was digested with restriction enzyme BstYI in 35 µl reaction volume for 2.5 h at 60° and denatured for 20 min at 80°. Synthetic top (Top-5′-nucleotide (nt)-1-GATCCCACTAGTGTCGACACCAGTCTCTAATTTTTTTTTTCAAAAAAA-nt-48-3′) and bottom splinkerette oligonucleotides (bottom-5′-nt-1-CGAAGAGTAACCGTTGCTAGGAGAGACCGTGGCTGAATGAGACTGGTGTCGACACTAGTGG-nt-61-3′) were annealed (10 mM each) with 10X NEB Buffer 2 (NEW EGLAND BioLabs) in H_2_O_2_ at 95° for 3 min. Digested genomic DNA (35 µl) was ligated to annealed splinkerette oligonucleotides (6 µl) with 1 µl of T4 DNA Ligase (NEW EGLAND BioLabs) in 50 µl of reaction volume at 15-16° for 16 h. The annealed product was directly subjected to first round PCR with a pair of primers, one targeting the bottom splinkerrette oligonucleotide (primer S1, nt 1-28) and the other one targeting *P{switch2}* (primer P1, 5′-nt-7818-CACTCAGACTCAATACGACAC-nt-7839-3′). First round PCR products (0.5 or 1 μl) were then used for second round PCR using another primer set, one targeting the bottom splinkerrette oligonucleotide (primer S2, nt 29-54), and the other targeting *P{swtch2}* (primer P2, 5′-nt-7888-GGATGTCTCTTGCCGAC-nt-7905-3′). Phusion High-Fidelity DNA Polymerase (New England BioLabs) or Taq DNA Polymerase 2X Master Mix RED (Ampliqon) were used for PCR reactions. Twenty microliters of second round PCR products were cleaned and sequenced with a primer targeting the 3′ end of *P{Switch}* (primer P3, 5′-nt 7904-CGGGACCACCTTATG-7918-nt-7918-3′).

Two microliters of purified second PCR products were ligated with pGEM vector (Promega) and transformed into JM109 High-Efficiency competent cells (Promega) for blue/white screening according to standard procedures. Plasmids were extracted using FavorPrep Plasmid Extraction Mini Kit (FAVORGEN) and sequenced by T7 or SP6 primer.

### Genomic PCR

Total genomic DNA was extracted from 10 flies using the DNeasy Blood & Tissue kit (QIAGEN) according to the instruction manual, or from 25 flies following the standard protocol provided by Vienna Drosophila RNAi Center. One microliter of genomic DNA was used for PCR using Tag DNA polymerase 2X Master Mix RED (Ampliqon) with a gene specific primer and a primer located on the 5′ region of *P{Switch*}, as listed below.

#1774:

*vri*-nt-16738-TCGTCGGAGAAATGCTTTTAC-nt-16758;
*P{Switch}*-nt-102-CACACAACCTTTCCTCTCAAC-nt-82

#2112:

*Mob2*-nt-8575-TCTGCTACTATTCTACTGCCAC-nt-8596;
*P{Switch}* nt-42-GCTTCGGCTATCGACGGGAC-nt-23

#2126:

*GstS1*-nt-1982-ACACACACAGTCAAACGCC-nt-2000
*P{Switch}*-nt-104-CGCACACAACCTTTCCTCTC-nt-85

#2261:

*Unc-115a*-nt-19-GTCGGTTTCGTTGCTGGTGC-nt-38;
*P{Switch}*-nt-42-GCTTCGGCTATCGACGGGAC-nt-23

#2305:

*EcR*-nt-14,616-CCCTCTTTGGTCGTCCGCAA-nt-14,635;
*P{Switch}*-nt-42-GCTTCGGCTATCGACGGGAC-nt-23

#2312:

*fax*-nt-96-GCTCCTGAATCCATCCGAATC-nt-116
*P{Switch}*-nt-102-CACACAACCTTTCCTCTCAAC-nt-82

### Data Availability

Strains are all available from the Drosophila fly stock centers, or upon request. The authors affirm that all data necessary for confirming the conclusions of the article are present within the article, figures, tables and supplementary information. Supplemental material available at Figshare: https://doi.org/10.25387/g3.8020115.

## Results and Discussion

### Generation of new GeneSwitch GAL4 lines that are expressed in the ovary

The GeneSwitch GAL4 protein is a GAL4-progestrone-receptor fusion, containing a GAL4 DNA-binding domain, a progesterone receptor and a NF-κB activation domain (Figure 1D). Activation of GeneSwitch GAL4 requires the binding of RU486 (a progesterone analog) to the progesterone receptor moiety (Osterwalder *et al.*, 2001; Roman *et al.*, 2001). Thus, precise control of transgene expression timing and levels can be achieved by feeding flies with food containing various doses of RU486.

To generate new lines with GeneSwitch GAL4 expression in the GSC-niche unit, we mobilized the enhancer-detector GeneSwitch GAL4, X-linked *{Switch2}19-2* by providing Transposase (*Δ*2-3). Flies bearing Transposase were recognized by the appearance of short bristles caused by the dominant *Stubble* (*Sb*) mutation. We first mobilized X-linked *{Switch2}19-2* from the *white* gene (w*) to the second chromosome, where the second chromosome balancer (*CyO*) was located (Figure 2A). The flies of the genotype *w*^-^; *{Switch2}19-2*, *CyO/+* displayed red eyes, long bristles and curly wings; these flies were collected and amplified after which jumping of the *{Switch2}19-2* was induced in order to generate new GeneSwitch GAL4 lines (Figure 2B). The male flies bearing new *{Switch2}19-2* insertions (red eyes with long bristles and without curly wings) were mated with female flies carrying the *UASp-gfp* transgene for 5 days. The progeny were then balanced by balancers following the standard crossing scheme to prevent recombination. One- or two-day-old *{Switch2}19-2>gfp* flies were fed with RU486 (10 µg/ml) for two days to induce GFP expression, and ovaries were dissected for GFP detection.

### Six GeneSwitch GAL4 lines show expression in germarial somatic cells

**Figure 3:**
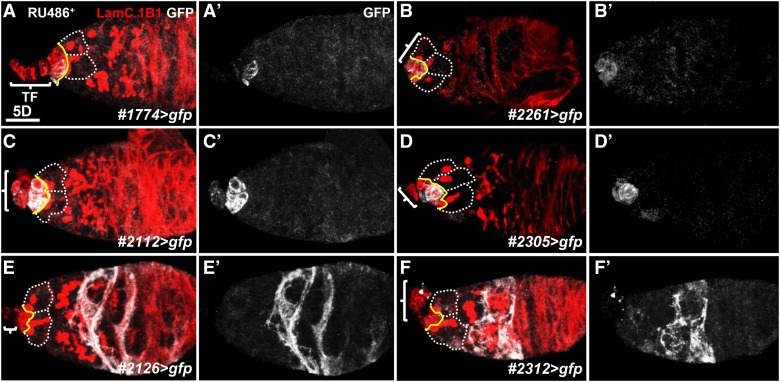
*GeneSwitch GAL4* lines carry drivers for GSC maintenance and differentiation niches. Five-day (D)-old germaria are shown from *GeneSwitch GAL4* (A) *#1774>gfp*, (B) *#2261>gfp*, (C) *#2112>gfp*, (D) *#2305>gfp*, (E) *#2126>gfp* and (F) *#2312>gfp* flies that were fed with RU486 (10 µg/ml) for two days. Germaria were stained with 1B1 (red, fusomes), LamC (red, terminal filament [TF] and cap cell nuclear envelope), and GFP (gray). (A’-F’) only show the GFP channel. White dashed lines encircle GSCs; yellow lines indicate the junction between GSCs and cap cells, and brackets indicate TFs. Scale bar is 10 µm.

We screened 2385 GeneSwitch GAL4 lines and found 79 lines (3.3% of the screened lines) had expression in ovarian somatic cells (Supplementary Table 1). Among them, 73 lines (3.1%) showed expression in the follicle cell lineage, and only six lines (0.25%) had expression in germarial somatic cells (Figure 3 and Table 1). GFP expression in lines #1774 and #2261 was present in cap cells and follicle cells (Figure 3A and B, and Supplementary Figure 1A-D). Line #2112 showed strong expression in cap cells but weaker expression in TFs (Figure 3C). Line #2305 had strong expression in cap cells and follicle cells (Figure 3D and Supplementary Figure 1E and F). Lines #2126 and #2312 showed expression in posterior escort cells (Figure 3E and F). Surprisingly, only one GeneSwitch GAL4 line (#2261) weakly expressed GFP in the germline (Table 1 and Supplementary Figure 1D). Given that 13% of *P[lacZ]*-mediated insertions (184 insertions) and 6.8% of *P[GFP-trap]*-mediated insertions (887 lines) were found to be expressed in the germline in previous studies (Fasano and Kerridge 1988; Hsu and Drummond-Barbosa 2017), we speculate that the low rate of germline expression in GeneSwitch GAL4 lines may be due to inefficient expression of GeneSwitch GAL4 at the transcriptional or translational levels. Indeed, a recent study has shown that many of our best genetic tools, including the GeneSwitch Gal4, bear hsp70 promoter, may be silenced by germline piRNAi (DeLuca and Spradling 2018).

**Table 1:** Ovarian Expression Patterns of GeneSwitch GAL4 Lines

Cell Type	Number
Germ cells	1 (#2261)
Cap cells/ TF	1 (#2112)
Cap cells/ Follicle cells	3 (#1774, #2261, #2305)
Escort cells	2 (#2126, #2312)
Follicle cells	73

### GeneSwitch GAL4 expression is inducible during aging in niche cap cells of lines #2261 and #2305 and escort cells of line #2312

**Figure 4:**
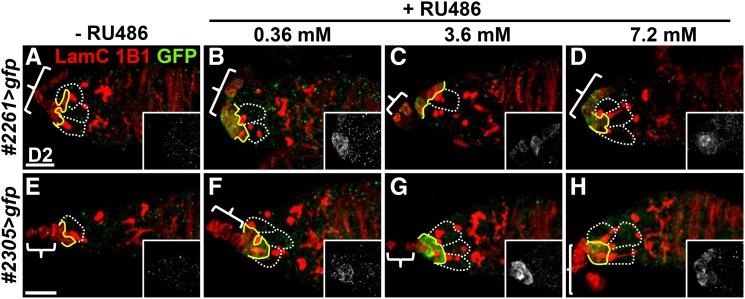
*GeneSwitch GAL4* lines are activated by different concentrations of RU486. (A-H) Two-day (D)-old *GeneSwith GAL4 #2261>gfp* (A-D) and #*2305>gfp* (E-H) germaria are shown from control flies (without RU486 feeding, A and E) and flies fed with 0.36 mM (B and F), 3.6 mM (C and G), and 7.2 mM RU486 (D and H) after eclosion. Germaria were stained with 1B1 (red; fusomes), Lam C (red; terminal filament [TF] and cap cell nuclear envelopes) and GFP (green). Scale bar is 10 μm. White dashed lines encircle GSCs; yellow lines indicate the junction between GSCs and cap cells, and brackets indicate TFs.

It has been shown that GeneSwitch GAL4 can be activated in flies fed for 2-4 days with a wide range of RU486 concentrations, from 0.023 µM (10 µg/ml, see Figure 2 and 3) to 500 mM (Ferris *et al.*, 2006; Poirier *et al.*, 2008; Sun *et al.*, 2017). However, aged flies eat less starting approximately 3 weeks after eclosion (Carey *et al.*, 2006), which may potentially reduce the levels of transgene expression due to lower RU486 uptake. To effectively induce transgene expression in aged flies, while avoiding potential RU486 toxicity, we administered a high concentration for only two days. We first fed young (2 to 3-day-old) *#2261>gfp* and *#2305>gfp* flies with wet yeast paste containing 0.36, 3.6 or 7.2 mM RU486 for two days and examined GFP expression in the ovary (Figure 4). Without RU486 feeding, GFP was not expressed in either *#2261>gfp* or *#2305>gfp* germaria (Figure 4A and E). Interestingly, the three different dosages of RU486 could all induce GFP expression in niche cap cells at similar levels (Figure 4B-D and F-H), but 3.6 mM RU486 induced the strongest GFP expression in #*2305>gfp* flies (Figure 4G). This result was consistent with a previous report that different GeneSwitch GAL4 drivers have different sensitivities to RU486 (Poirier *et al.*, 2008). In addition, the concentration of RU486 we used did not strongly affect oogenesis, according to an egg production assay (Supplementary Figure 2).

**Figure 5:**
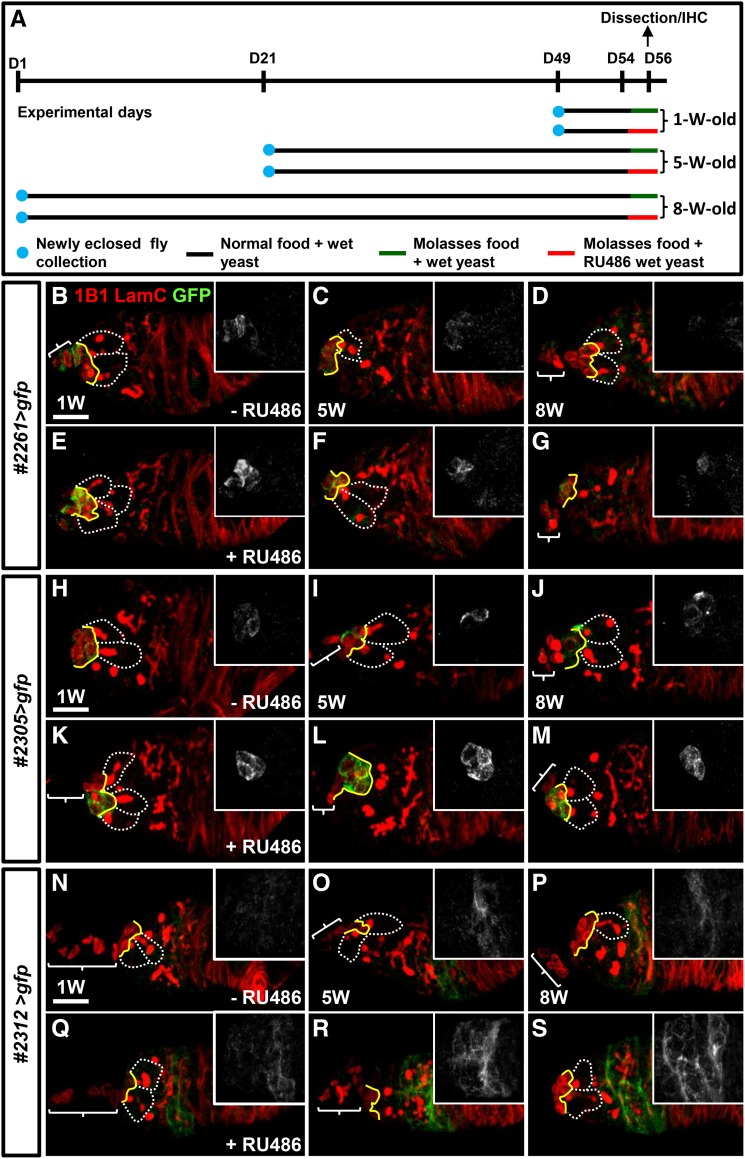
The *GeneSwitch GAL4* lines retain expression in aged germaria. (A) The scheme for examining expression of *GeneSwitch GAL4* in germaria of 1-, 5-, and 8-week (W)-old flies. Newly eclosed flies carrying *GeneSwitch GAL4>UASp-gfp* were collected at experimental days 1, 21 and 49, and respectively cultured until the ages of 8-, 5-, and 1-week-old, prior to dissection for immunohistochemistry (IHC). Flies were maintained with standard media (normal food + wet yeast) until two days before dissection; at experimental day 54, flies were switched to a molasses-agar medium plus a wet yeast paste with or without RU486 for two days. Food was changed daily until dissection. (B-S) One- (B, E, H, K, N and Q), 5- (C, F, I, L, O and R) and 8-week-old (D, G, J, M, P and S) *GeneSwitch GAL4 #2261>gfp* (B-G), *#2305>gfp* (H-M), and *#2312>gfp* (N-S) flies were fed with wet yeast (B-D, H-J, and N-P) or wet yeast containing RU486 (E-G, K-M, and Q-S) for two days prior to dissection. Germaria were stained with 1B1 (red; fusomes), Lam C (red; terminal filament [TF] and cap cell nuclear envelopes) and GFP (green). Inserts only show the GFP channel (gray). White dashed lines encircle GSCs; yellow lines indicate the junction between GSCs and cap cells, and brackets indicate TFs. Scale bar is 10 µm.

We next proceeded to test if expression of the six germarial cell GeneSwitch GAL4 lines were affected by aging. To avoid staining variation, we collected newly eclosed *GeneSwitch GAL4>gfp* flies at experimental day 1, 21 and 49 and, cultured them until they reached 8 weeks, 5 weeks and 1 week of age, respectively (Figure 5A). The flies were maintained on a normal diet with wet yeast paste until experimental day 54, and then were switched to molasses agar (to maintain moisture) plus wet yeast paste with or without 3.6 mM RU486 for two days. Ovaries of different aged flies were dissected at experimental day 56 and examined for GFP expression. We did not select lines #1774, #2112 and #2312 for the GSC aging study because their expression characteristics did not match our current goals (Supplementary Figure 3). Most *#1774>gfp* germaria weakly expressed GFP in only one niche cap cell (Supplementary Figure 3 A-F), while expression of GFP in niche cap cells of *#2112>gfp* flies was not observed in 5-week-old flies (Supplementary Figure 3 G-L). Furthermore, line #2126 had expression in escort cells of 8-week-old flies in the absence of RU486 supplementation (Supplementary Figure 3 M-R). In contrast, expression of the GeneSwitch niche (lines #2261 and #2305) and escort cell drivers (#2312) were robust and retained during aging (Figure 5 B-S). These three lines all expressed low levels of GFP in the absence of RU486, and GFP expression was much stronger in the presence of RU486. These results suggest that these three lines may be useful tools to study GSC aging.

### Overexpression of Dilp2 in the aged niche delays GSC loss

It has been previously proposed that insulin signaling controls the size of the ovarian GSC niche to maintain GSCs during aging (Hsu and Drummond-Barbosa 2009). Cap cell number is decreased in the niche of *Insulin Receptor* mutant and aged flies, while overexpression of Dilp2 in the niche using the traditional *GAL4/UAS* system throughout adult life delays age-dependent loss of GSCs and niche cells (Hsu and Drummond-Barbosa 2009). However, continuous overexpression of Dilp2 may not only help niche cap cells to resist the effects of aging, but it could also enhance function of non-aged niche cells. To understand the requirement for Dilp2 for GSC maintenance specifically in the aged niche, we induced Dilp2 expression in aged *#2261>dilp2* and *#2305>dilp2* females for two days and examined the number of GSCs. GSCs were unambiguously identified by their anteriorly anchored fusomes (Xie and Spradling 2000). *#2261>dilp2* and *#2305>dilp2* females were maintained on a standard medium with a wet yeast paste until experimental day 5, 33 and 54, and then switched to a molasses plate with wet yeast paste with or without 3.6 mM of RU486 for two days (See Figure 5A).

**Figure 6:**
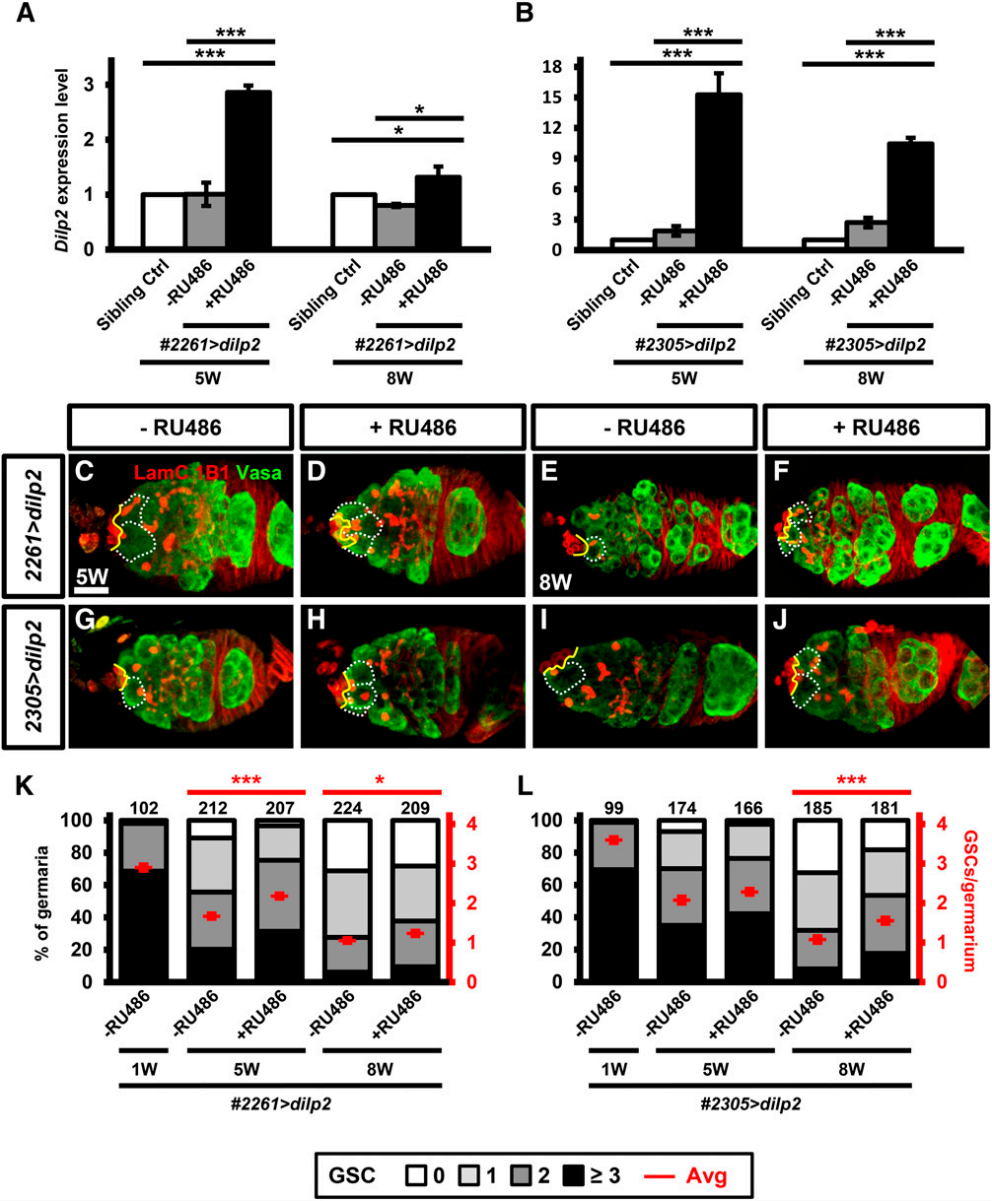
Overexpression of Dilp2 in the aged niche delays GSC loss. (A and B) Dilp2 expression levels of #*2261>dilp2* (A), *#2305>dilp2* (B) and their sibling controls (Sibling Ctrl) at 5- and 8-week (W)-old by three independent qPCR trials. #*2261>dilp2* and *#2305>dilp2* flies without or with RU486 (3.6 mM) feeding two days before reaching ages of 5- and 8-week old; sibling control flies were maintained on a standard media until dissection. *, *P* < 0.05; **, *P* < 0.01; ***, *P* < 0.001. Error bars are standard deviation. (C to J) 5- (C, D, G, H) and 8-week-old (E, F, I, J) *GeneSwitch GAL4 #2261>dilp2* (C-F), *#2305>dilp2* (G-J), germaria of flies fed without (C, E, G, I,) or with RU486 (D, F, H, J,) two days before dissection to activate Dilp2 expression. Germaria were stained with 1B1 (red; fusomes), Lam C (red; terminal filament (TF) and cap cell nuclear envelopes) and Vasa (green, germ cells). White dashed lines encircle GSCs; yellow lines indicate the junction between GSCs and cap cells, and brackets indicate TFs. Scale bar, 10 µm. (K and L) GSC numbers in #*2261>dilp2* (K), *#2305>dilp2* (L) at 1, 5, and 8 weeks. Flies of indicated genotypes were fed without or with RU486. The left Y-axis shows percentage (%) of germaria carrying 0, 1, 2, 3 or more than 3 GSCs, and the right Y-axis shows average (avg.) number of GSCs per germarium. Number of germaria analyzed are shown above each bar. *, *P* < 0.05; **, *P* < 0.01; ***, *P* < 0.001. Error bars are SEM. Data were analyzed by Student’s *t*-test according to average number of GSCs per germarium.

We first used qRT-PCR to examine whether two days of RU486 feeding was able to increase *dilp2* transcripts in the aged ovaries. Unfortunately, we were not able to examine Dilp2 protein levels due a lack of available anti-Dilp2 antibody. Nevertheless, we found that *dilp2* expression levels were significantly increased in 5- and 8-week-old *#2261>dilp2* and *#2305>dilp2* flies after two days of RU486 feeding, compared to flies without RU486 feeding and sibling controls (Figure 6A and B). Noticeably, *dilp2* expression levels in 5- and 8-week-old *#2305>dilp2* flies with two-day RU486 feeding were fivefold and eightfold higher, respectively, than those in age-matched *#2261>dilp2* flies fed with RU486. Thus, line #2305 appears to be a stronger driver than line #2261 (see also Figure 3, 4 and 5). Surprisingly, two days of RU486 feeding was able to delay GSC loss in aged flies from the two lines. In *#2261>dilp2* flies without RU486 feeding, aged germaria carried significantly lower number of GSCs compared to young germaria (1-week (W): 2.9 ± 0.1 GSCs, n = 102 germaria; 5W: 1.7 ± 0.1 GSCs, n = 212 germaria, *P* < 0.001, 8W: 1.1 ± 0.1 GSCs, n = 224 germaria, *P* < 0.001) (Figure 6C-F and K). These results are in agreement with previous reports that aging reduces GSC number. Compared to *#2261>dilp2* without RU486 feeding, 5W and 8W *#2261>dilp2* flies with 2 days RU486 feeding carried significantly higher numbers of GSCs (5W: 2.2 ± 0.1 GSCs, n = 207, *P* < 0.001; 8W: 1.2 ± 0.1 GSCs, n = 209, *P* < 0.05). Similarly, germaria of *#2305>dilp2* flies without RU486 feeding carried 2.1 ± 0.1 GSCs (n = 174) and 1.1 ± 0.1 GSCs (n = 185) at 5W and 8W (Figure 6G-J and L), respectively. *#2305>dilp2* flies at 5W with 2 days RU486 feeding carried a higher number of GSCs (2.3 ± 0.1 GSCs, n = 181), but the difference did not reach statistical significance compared to *#2261>dilp2* without RU486 feeding (*P* = 0.067). However, 8W *#2305>dilp2* flies with 2 days RU486 feeding carried significantly higher number of GSCs (1.6 ± 0.1 GSCs, n = 166), as compared to those without RU486 feeding (*P* < 0.001). Increased duration of RU486 feeding (5 days) only slightly enhanced GSC maintenance (5W: 2.1 ± 0.1 GSCs, n = 70 to 2.6 ± 0.1 GSCs, n = 94. *P* < 0.001; 8W: 1.1 ± 0.1 GSCs, n = 98 to 1.4 ± 0.1 GSCs, n = 97. *P* < 0.05) (Supplementary Figure 4) but also resulted in more germ cell cysts within the germaria and the occurrence of two side-by-side egg chambers in the ovariole string or abnormal morphology of germaria that caused difficulty to count GSCs (Supplementary Figure 5). Nevertheless, our results confirm that the GeneSwitch GAL4 lines we generated are suitable to be used for studying GSC aging.

### Identification of the germarial somatic cell-expressed genomic loci that were integrated by GeneSwitch GAL4

**Figure 7:**
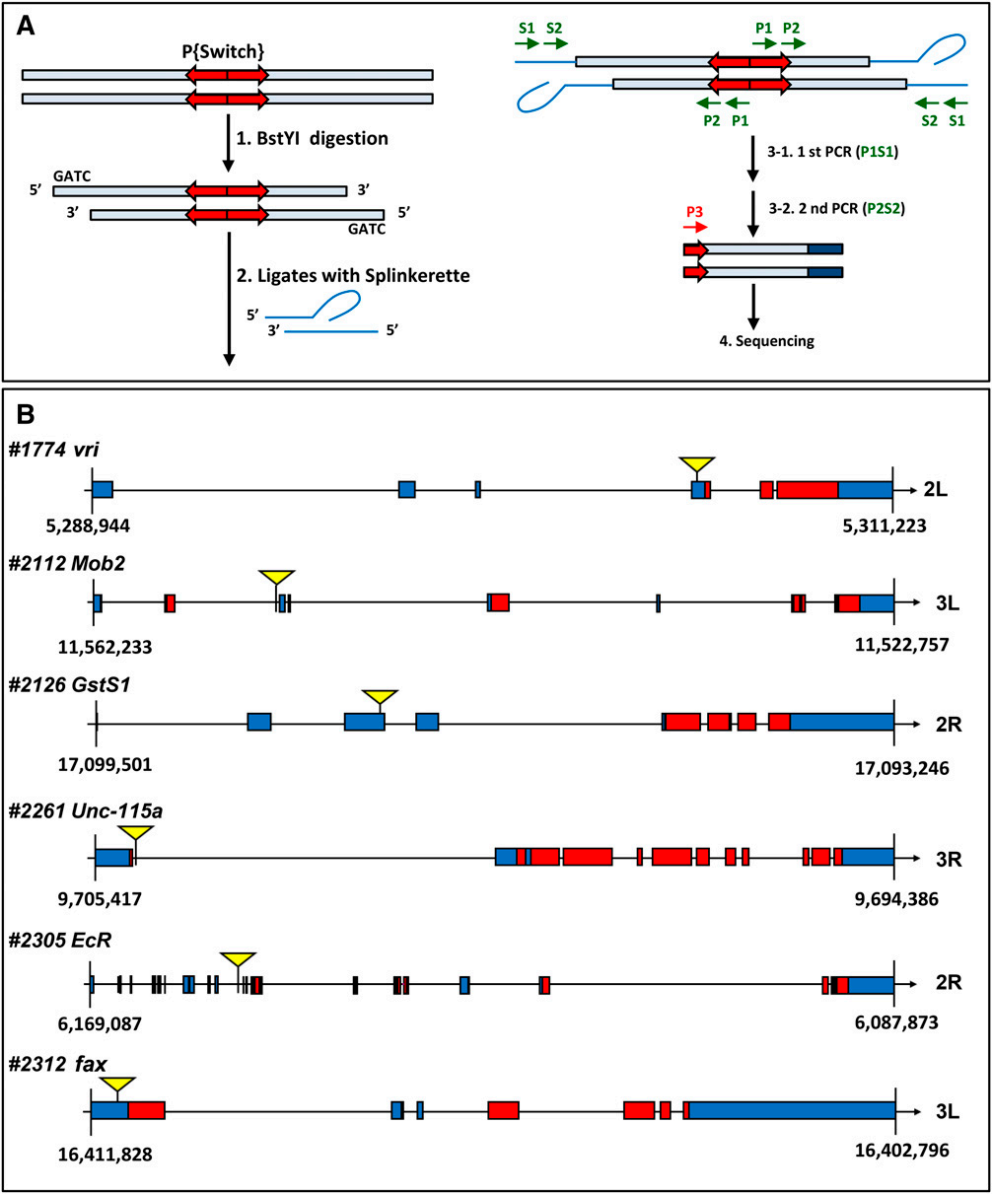
Identification of the *P{Switch}* integration site. (A) Diagram of the procedure for mapping the *P{Switch}* integrations in the genome by splinkerette PCR. Genomic DNA was first digested with BstYI and subsequently ligated to the splinkerette linker. The genomic DNA-GeneSwitch GAL4 junctions were amplified in two rounds of PCR for sequencing. P, transposon primers; S, splinkerette primers. (B) Schematic showing the genomic loci of the mapped *GeneSwitch GAL4 #1774*, *#2112*, *#2126*, *#2261*, *#2305*, and *#2312* within the *vri*, *Mob2*, *GstS1*, *Unc-115a*, *EcR*, and *fax* genes, respectively. The numbers 2 and 3 denote chromosome number; L, left arm; R, right arm. Arrows indicate gene orientation. Numbers below gene structures represent genomic location of a gene. Blue boxes indicate untranslated regions, and red boxes indicate coding sequences.

To identify the genomic insertion site of the *P{Switch2}* lines that exhibited expression in niche cap cells and escort cells of the germarium, we carried out splinkerette PCR for sequencing (Figure 7A) (Uren *et al.*, 2009), which is more efficient and simpler than inverse PCR (Potter and Luo 2010). Our results showed that *P{Switch2}* was inserted into the fourth exon of *vrille* (*vri*) on the left arm of the second chromosome (2L) in line *#*1774. The insertion was in the ninth intron of *Mps1-one-binder 2* (*Mob2*) on the left arm of the third chromosome (3L) in line *#*2112. The construct was inserted into the sixth exon of *Glutathione S transferase 1* (*GstS1*) on 2R in line *#*2126. The insertion was in the last intron of *Uncoordinated 115a* (*Unc-115a*) on the right arm of the third chromosome (3R) in line *#*2261. *P{Switch2}* was inserted into the sixteenth intron of *Ecdysone Receptor* (*EcR*) on the right arm of the second chromosome (2R) in line *#*2305, and finally, the insertion was in the last exon of *failed axon connections* (*fax*) on 3L in line *#*2312 (Figure 7B). We validated these results by subcloning splinkerette PCR products into the TA vector for sequencing, or by using a locus-specific primer with a primer from the *P{Switch2}*.

*P{vri^1774^-Switch2}*, *P{EcR^2305^-Switch2}*, *P{Unc-115a^2261^-Switch2}* and *P{Mob2^2112^-Switch2}* are expressed in niche cap cells (see Figure 3), suggesting Vri, EcR, Unc-115a, and Mob2 may be involved in GSC maintenance via niche formation or function. *vri* encodes a basic leucine zipper transcription factor (George and Terracol 1997) and acts as an enhancer of *dpp* phenotypes both in embryos and developing wings (George and Terracol 1997). Niche cap cells produce Dpp as a stemness factor to maintain GSC identity (Xie and Spradling 1998). Together, these findings are highly suggestive that Vri is involved in GSC maintenance. EcR, a receptor for ecdysteroids (Koelle *et al.*, 1991), coordinates behavioral, genetic and morphological changes in *Drosophila* tissue development (Buszczak *et al.*, 1999; Gaziova *et al.*, 2004; Kozlova and Thummel 2003; McBrayer *et al.*, 2007). It has been previously shown that EcR controls niche size during ovary development (Hitrik *et al.*, 2016; Morris and Spradling 2012); however, the role of EcR in the adult niche remains unclear. Unc-115a has a villin-headpiece domain that binds to actin (Garcia *et al.*, 2007), and it regulates axon projections in the central nervous system (Garcia *et al.*, 2007; Roblodowski and He 2017). Niche cap cells are enriched in F-actin filament (data not shown), suggesting a role for Unc-115a in determining niche architecture. Mob2 belongs to the conserved Mob1/phocein domain protein family (He *et al.*, 2005), and it controls photoreceptor development (Liu *et al.*, 2009) and synaptic growth at the neuromuscular junction (Campbell and Ganetzky 2013). In addition, yeast Mob2 was found to promote polarized cell growth and to induce asymmetric cell fate (Colman-Lerner *et al.*, 2001) as well as cell morphogenesis (Hou *et al.*, 2003). However, the role of Mob2 in the GSC niche remains unclear (Campbell and Ganetzky 2013; Liu *et al.*, 2009).

*P{GstS1-Switch2}* and *P{fax-Switch2}* are expressed in escort cells (see Figure 3), which control germ cell proliferation and differentiation (Eliazer *et al.*, 2014; Kirilly *et al.*, 2011; Su *et al.*, 2018). GstS1 is a Glutathione S-transferase (Beall *et al.*, 1992), which eliminates hydrogen peroxide and catalyzes the conjugation of reduced glutathione to oxidized substrates for the purpose of detoxification. It has been previously proposed that redox status in escort cells influences germ cell differentiation (Wang *et al.*, 2015), suggesting that GstS1 may function in this context. Fax contains Gst N-and C-terminal domains, and the protein is known to promote actin remodeling in the formation of axonal connections during development (Hill *et al.*, 1995; Liebl *et al.*, 2000) as well as membrane extension of escort cells for germ cell development in response to insulin signaling (Su *et al.*, 2018). However, the functions of Gst N- and C-terminal domains in the Fax protein are not clear. Overall, the results of our GeneSwitch screen provide promising tools for studying aging, and at the same time, our results identify genes that may function to control homeostasis of GSCs and their progeny to promote functional reproduction.
